# Case Report: Inferior Bilobectomy for Lung Cancer to Allow Weaning From Veno-Venous Extracorporeal Membrane Oxygenation

**DOI:** 10.3389/fsurg.2021.736541

**Published:** 2021-09-21

**Authors:** Francesca Signore, Debora Brascia, Marcella Schiavone, Giulia De Iaco, Teodora Panza, Angela De Palma, Francesco Murgolo, Antonio Civita, Rosa Di Mussi, Salvatore Grasso, Giuseppe Marulli

**Affiliations:** ^1^Thoracic Surgery Unit, Department of Organ Transplantation and Emergency, University Hospital of Bari, Bari, Italy; ^2^Anesthesia and Intensive Care Unit, Department of Organ Transplantation and Emergency, University Hospital of Bari, Bari, Italy

**Keywords:** veno-venous extracorporeal membrane oxygenation, respiratory failure, lung cancer, lung resection, multidiscipinary approach

## Abstract

In recent years, veno-venous extracorporeal membrane oxygenation (V-V ECMO) has allowed complex lung and airways resections in patients with a compromised perioperative respiratory function. In the following report, we present a case of successful weaning from V-V ECMO in a patient who underwent an inferior bilobectomy for lung cancer.

## Introduction

Veno-venous extracorporeal membrane oxygenation (V-V ECMO) has been widely used as rescue therapy for refractory acute respiratory failure since the 1970s (1). In recent years, its applications have spread in the field of thoracic surgery allowing complex resections in patients with a compromised perioperative respiratory function, who would be otherwise excluded from surgery.

We present a case of a patient admitted to the Hospital respiratory ECMO unit for acute refractory hypoxemia, requiring V-V ECMO, successfully weaned from ECMO, and subsequently discharged from the intensive care, after an inferior bilobectomy.

## Case Description

**Figure 1:**
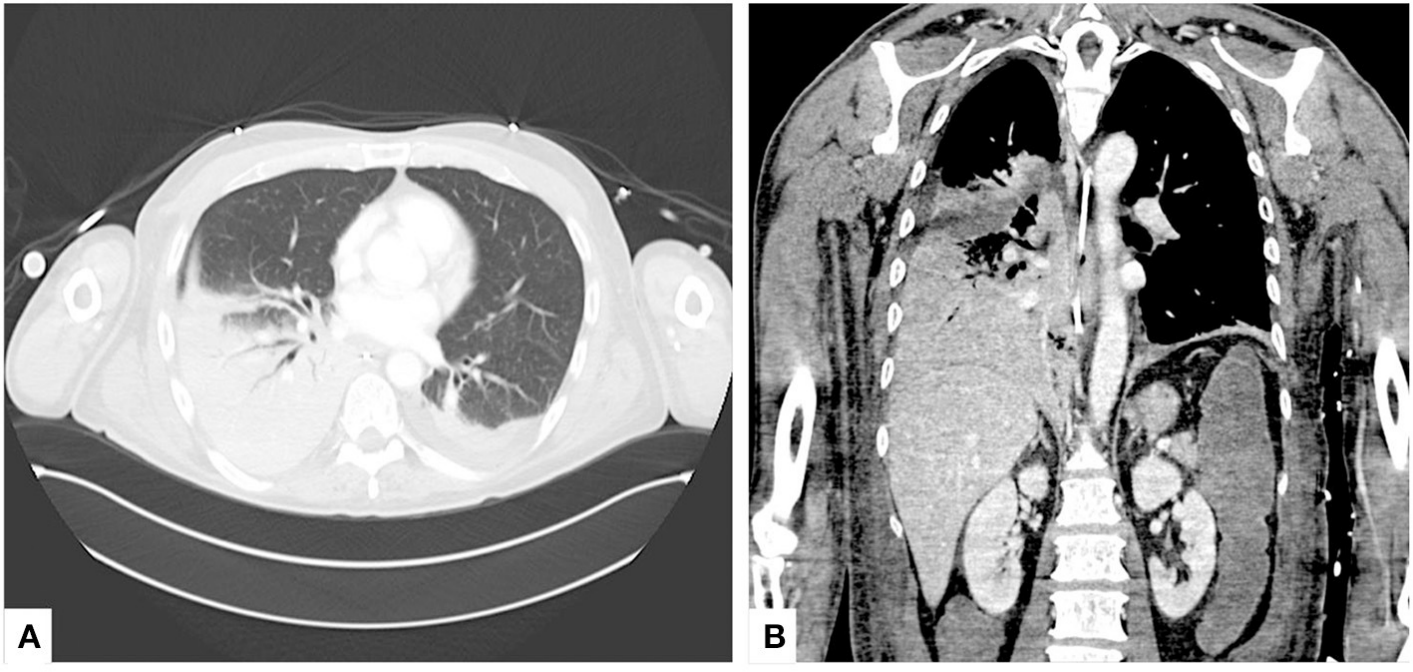
Preoperative CT scan. **(A)** Axial and **(B)** coronal views of admission chest CT scans showing mixed ground-glass opacities and consolidation with positive air bronchograms involving both the medium and the lower right lobes.

A 60-year-old man was admitted to the Pneumology Unit with complaints of worsening dyspnoea and peripheral cyanosis. Chest X-ray revealed pulmonary consolidations with right basal predominance. The medical history was uneventful for previous chronic respiratory failure. Initial empirical therapy with broad-spectrum antimicrobials was started, even in the absence of the common clinical or laboratory signs of pneumonia. On the 4th day of hospitalization, oxygen therapy by Venturi Mask was ineffective and the patient worsened developing severe hypoxemia (paO_2_ 48 mmHg, SpO_2_ 82%); for that reason, non-invasive bilevel positive pressure ventilation was started (BiPAP - IPAP 10 cmH_2_O and EPAP 6 cmH_2_O, FiO_2_ 90%), leading to similar poor results (SpO_2_ 84%, pH 7.47, pCO_2_ 37 mmHg, paO_2_ 51 mmHg) and, thus, the patient was submitted to endotracheal intubation and admitted to intensive care unit (ICU). Chest computed tomography scan revealed mixed ground-glass opacities and consolidation with positive air bronchograms involving both the medium and the lower right lobes (Figure 1). The whole panel for community-acquired pneumonia did not show any positive results and, accordingly, a cytological examination of bronchial washing was sent in the suspicion of a neoplastic nature of the lesions. Despite lung-protective mechanical ventilation and pronation, according to the current protocols (2), the respiratory condition did not improve and the patient eventually presented refractory hypoxemia on day 5 from ICU admission. Accordingly, an urgent decision was made to initiate V-V ECMO (CardioHelp, Getinge Critical Care, Sweden), by percutaneous cannulation of the right femoral vein (25-Fr draining cannula) and the right jugular vein (17-Fr restitution cannula). At the start of the ECMO run, blood flow was set at 4 liters per min (lpm), sweep gas flow at 2 L/min and unfractionated heparin was infused to match a PTT ratio 1.5 to 2-folds the baseline. Ultra-protective mechanical ventilation was instituted in Controlled Constant Pressure Mode (PCV) mode with a driving pressure of 8 cmH_2_O, Peep 12 cmH_2_O, RR 8, FiO_2_ 0.6.

Two days after the start of V-V ECMO, the results of the bronchial washing revealed an adenocarcinoma. On the next day, the patient was extubated and left in spontaneous breathing supported by High Flow Nasal Cannula (HFNC) oxygen therapy at 60 L/min with a FiO_2_ of 60% (awake ECMO). In the following days, the patient remained fully dependent on ECMO as the gas exchange critically worsened at any ECMO weaning attempt. After an interdisciplinary evaluation of the general conditions and oncologic history of the patient, it was supposed that the right inferior consolidation had led to intrapulmonary shunting which was the main cause of hypoxemia and poor gas exchanges. V-V ECMO-assisted surgical resection was, therefore, planned based on both oncological and physio-pathological reasons. On day 18 after ICU admission, an inferior bilobectomy with systematic lymphadenectomy was performed through a right lateral thoracotomy. Of note, during surgery, we documented a sudden improvement of arterial oxygenation immediately after the right main pulmonary artery clamping, confirming the hypothesis that an extensive shunt occurred within the adenocarcinoma. Final histopathologic analysis revealed multifocal lung adenocarcinoma with the invasion of the visceral pleura (T4N0M0). The heparin infusion for the V-V ECMO support was stopped 4 h prior to the operation and throughout the surgical time; heparin infusion was resumed the next day.

**Figure 2:**
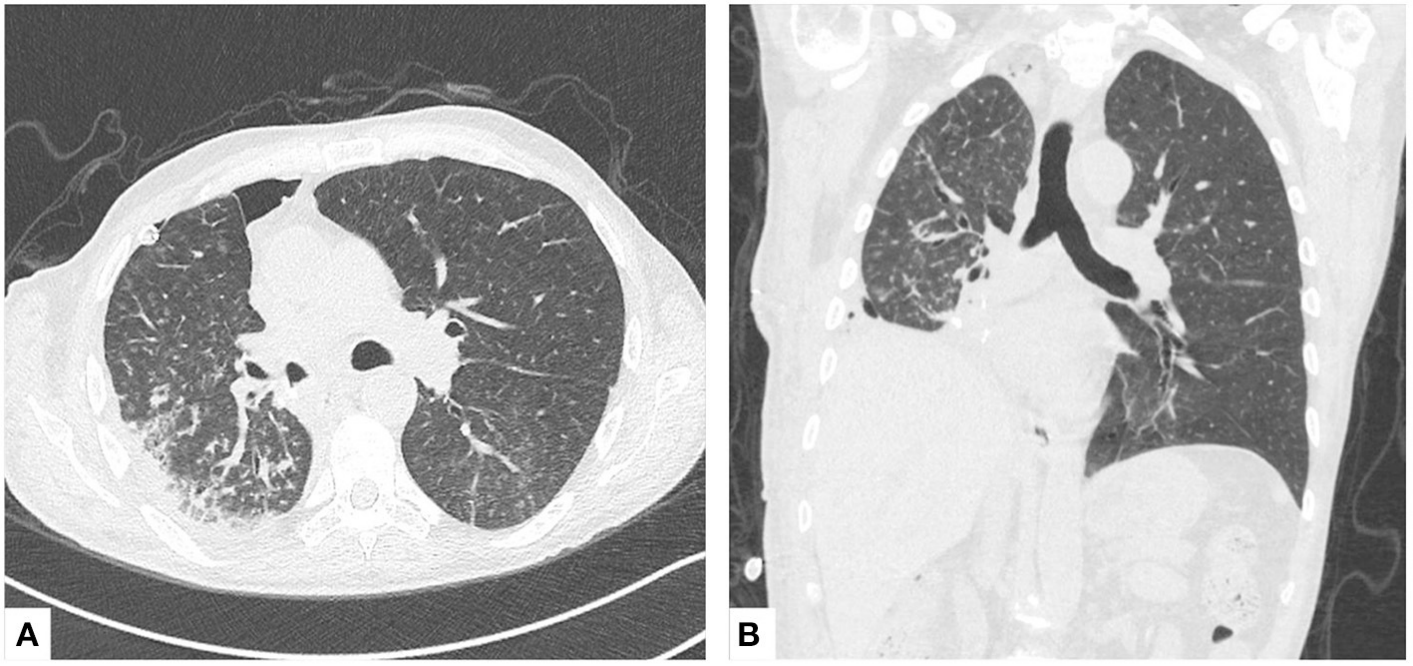
Postoperative CT scan. **(A)** Axial and **(B)** coronal views of chest CT scans 1 month after the inferior bilobectomy.

On the third postoperative day, after 16 days since its start, the patient was successfully weaned from V-V ECMO. On the eighth postoperative day, the patient was transferred to the thoracic surgery ward. The postoperative course was characterized by fever, persistent hyponatremia, and dyspnoea requiring few days of NIV. The patient was discharged on the 18th postoperative day. One month after the operation, he had no requirement for additional oxygen, and a postoperative CT scan demonstrated a sufficiently expanded residual right upper lobe with peripheral tree-in-bud and ground glass areas (Figure 2). Three months after surgery, six cycles of cisplatin and pemetrexed were administered as adjuvant chemotherapy regimen, followed by three cycles of consolidation pemetrexed. A disease relapse on both lungs was observed 7 months later and death occurred 1 year after surgery because of disease progression.

## Discussion

Veno-venous extracorporeal membrane oxygenation has been used as rescue therapy for the most severe cases of respiratory failure since the 1970s, and its applications have widely spread in the last 20 years, especially in the transplantations field. The use of V-V ECMO in thoracic surgery, other than lung transplantation, has been first described in 1996 by Horita et al. (3) who performed two successful resections and reconstruction of the carina under V-V-ECMO. The extracorporeal membrane oxygenation is of paramount importance in complex ventilatory situations, both allowing good haematosis and good surgical exposure, but its use has been only described in few case reports, small case series, and reviews (4–10). V-V ECMO should be considered in patients with limited pulmonary reserve, who had previously undergone pulmonary surgery and who need contralateral resections for second malignancies, in the lifesaving emergency treatment of massive hemoptysis, in the treatment of large mediastinal masses which cause compression of the trachea or the great vessels, in complex tracheobronchial resections and reconstructions, and in the resection of locally advanced tumors invading the heart and the great vessels (8–12). Lang et al. (10) reported a case series of nine patients who underwent complex tracheobronchial and greater thoracic vessels resections for lung cancer under V-V ECMO support, allowing an R0 resection in almost all cases (89%) and a 5-years survival of 76.7%. Redwan et al. (11) reported two cases in which the use of V-V ECMO allowed 40 min of apnea, enough to perform lung resections for carcinoma in patients with severely impaired preoperative pulmonary function. In all these cases, however, perioperative V-V ECMO was planned preoperatively, whereas in the present case, the surgical decision was made after a multidisciplinary discussion in a patient that had been urgently submitted to a V-V ECMO run for refractory, immediately life-threatening hypoxemia likely caused by the wide right to left blood shunt occurring within the tumor mass to both resect the carcinoma and to wean off ECMO. Indeed, according to the clinical reasoning, the surgical removal of perfused but unventilated atelectatic areas, commonly found in lung carcinoma, promptly reversed refractory hypoxemia allowing to discontinue V-V ECMO and successfully discharge the patient from the ICU and subsequently from the hospital (13).

In conclusion, we report on a complex case successfully managed through a multidisciplinary approach. Lung resection surgery during V-V ECMO allowed resolving a complex ethical and clinical issue in a patient that very likely would have to be not submitted to V-V ECMO if the neoplastic origin of his refractory hypoxemia would have been known before cannulation.

## Ethics Statement

Written informed consent was obtained from the relevant individuals' legal guardian/next of kin for the publication of any potentially identifiable images or data included in this article.

## Author Contributions

FS and DB: collected information of the patient, revised the literature and drafted the manuscript. MS, ADP, and GM: performed the operation. GDI and TP: revised the literature. FM, AC, and RDM: provided the anestesiological support during the operation. SG and GM: revised and supervisioned the writing of the manuscript. All authors contributed to the article and approved the submitted version.

## Conflict of Interest

The authors declare that the research was conducted in the absence of any commercial or financial relationships that could be construed as a potential conflict of interest.

## Publisher's Note

All claims expressed in this article are solely those of the authors and do not necessarily represent those of their affiliated organizations, or those of the publisher, the editors and the reviewers. Any product that may be evaluated in this article, or claim that may be made by its manufacturer, is not guaranteed or endorsed by the publisher.
